# Supplementary material for: Virtual Extensive Read-Across: A New Open-Access Software for Chemical Read-Across and Its Application to the Carcinogenicity Assessment of Botanicals
Source: Molecules. 2022 Oct 5;27(19):6605. doi: 10.3390/molecules27196605 (PMC9570968; doi:10.3390/molecules27196605)
Supplement: Supplementary file 1 [file molecules-27-06605-s001.zip › supplementary/molecules-1903480-supplementary1.pdf]

Molecular groups (MGs) for carcinogenicity

SMARTS of RDKit MGs	Description
[CX3]=[OX1]	Number of carbonyl O
[C!$(C-[OH])]=O	Number of carbonyl O, excluding COOH
[C!$(C=O)]-[OH]	Number of aliphatic hydroxyl groups
c[OH1]	Number of aromatic hydroxyl groups
[OX2](-[#6])-[CH3]	Number of methoxy groups -OCH3
[CX3]=[NX2]-[OX2]	Number of oxime groups
[#6][CX3](=O)[OX2H0][#6]	Number of esters
C-C(=O)[O;H1,-]	Number of aliphatic carboxylic acids
c-C(=O)[O;H1,-]	Number of Aromatic carboxylic acide
[#6]C(=O)[O;H,-1]	Number of carboxylic acids
[CX3](=O)[OX1H0-,OX2H1]	Number of carboxylic acids
[#6][CX3](=O)[#6]	Number of ketones
[OD2]([#6])[#6]	Number of ether oxygens (including phenoxy)
[OX2H]-c1ccccc1	Number of phenols
[CX3H1](=O)[#6]	Number of aldehydes
[$([NX4+]),$([NX4]=*)]	Number of quarternary nitrogens
[NH2,nH2]	Number of Primary amines
[NH1,nH1]	Number of Secondary amines
[NH0,nH0]	Number of Tertiary amines
n	Number of aromatic nitrogens
[nH]	Number of aromatic amines
c-[NX3;!$(N=*)]	Number of anilines
[Nv3](=C)-[#6]	Number of Imines
[NX1]#[CX2]	Number of nitriles
[NX3]-[NX3]	Number of hydrazine groups
C=N-[NX3]	Number of hydrazone groups
[N!$(N-O)]=O	Number of nitroso groups, excluding NO2
[N!$(N=O)](-O)-C	Number of hydroxylamine groups
[$([NX3](=O)=O),$([NX3+](=O)[O	Number of nitro groups
[#6]-N=N-[#6]	Number of azo groups
[N+]#N	Number of diazo groups
[$(*-[NX2-]-[NX2+]#[NX1]),$(*-[N	Number of azide groups
C(=O)-N	Number of amides
C(=O)-[NH2]	Number of primary amides
C(=N)(-N)-[!#7]	Number of amidine groups
C(=N)(N)N	Number of guanidine groups
[nH]	Number of H-pyrrole nitrogens
N(-C(=O))-C=O	Number of imide groups
N=C=O	Number of isocyanates
N=C=S	Number of isothiocyanates
S-C#N	Number of thiocyanates
[#9,#17,#35,#53]	Number of halogens
[CX4]-[Cl,Br,I,F]	Number of alkyl halides
[SX2](-[#6])-C	Number of thioether
[SH]	Number of thiol groups
C=[SX1]	Number of thiocarbonyl
S(=,-[OX1;+0,-1])(=,-[OX1;+0,-1])(	Number of sulfone groups
S(=,-[OX1;+0,-1])(=,-[OX1;+0,-1])(	Number of sulfone groups
N-S(=,-[OX1;+0,-1])(=,-[OX1;+0,-1	Number of sulfonamides
[NH2]-S(=,-[OX1;+0;-1])(=,-[OX1;	Number of primary sulfonamides
C1C(=O)NC(=O)NC1=O	Number of barbiturate groups
C(=O)(-N)-N	Number of urea groups
C#[CH]	Number of terminal acetylenes
n1cncc1	Number of imidazole rings
o1cccc1	Number of furan rings
s1cccc1	Number of thiophene rings
c1scnc1	Number of thiazole rings
c1ocnc1	Number of oxazole rings
n1ccccc1	Number of pyridine rings
N1CCCCC1	Number of piperdine rings
N1CCNCC1	Number of piperzine rings
O1CCNCC1	Number of morpholine rings
N1C(=O)CC1	Number of beta lactams
[C&R1](=O)[O&R1][C&R1]	Number of cyclic esters (lactones)
c1nnnn1	Number of tetrazole rings
O1CC1	Number of epoxide rings
[R0;D2][R0;D2][R0;D2][R0;D2]	Number of unbranched alkanes of at least 4 members (excludes halogenated alkanes)
[R2][R2]	Bicyclic
c1ccccc1	Number of benzene rings
[$(P(=[OX1])([$([OX2H]),$([OX1-<br>]),$([OX2]P)])([$([OX2H]),$([OX<br>1-<br>]),$([OX2]P)])[$([OX2H]),$([OX1-<br>]),$([OX2]P)]),$([P+]([OX1-<br>])([$([OX2H]),$([OX1-<br>]),$([OX2]P)])([$([OX2H]),$([OX<br>1-<br>]),$([OX2]P)])[$([OX2H]),$([OX1-<br>]),$([OX2]P)])]	Number of phosphoric acid groups
[$(P(=[OX1])([OX2][#6])([$([OX2<br>H]),$([OX1-<br>]),$([OX2][#6])])[$([OX2H]),$([O<br>X1-<br>]),$([OX2][#6]),$([OX2]P)]),$([P+<br>]([OX1-<br>])([OX2][#6])([$([OX2H]),$([OX1-<br>]),$([OX2][#6])])[$([OX2H]),$([O<br>X1-]),$([OX2][#6]),$([OX2]P)])]	Number of phosphoric ester groups
[$(c1(-[$([NX3](=O)=O),$([NX3+]	Number of nitro benzene ring substituents
[$(c1(-[$([NX3](=O)=O),$([NX3+]	Number of non-ortho nitro benzene ring substituents
[$([NX3H1]1-C=C-C-C=C1),$([Nv3	Number of dihydropyridines
[$(c1(-[OX2H])ccccc1);!$(cc-!:[CH	Number of phenolic OH excluding ortho intramolecular Hbond substituents
[$(C-[OX2H]);!$([CX3](-[OX2H])=	Number of aliphatic hydroxyl groups excluding tert-OH
[c&R2]12[c&R1][c&R1][c&R1][c&	Number of benzodiazepines with no additional fused rings
[$([cH]1[cH]cc(c[cH]1)~[$([#8,$([	Number of para-hydroxylation sites
[$(C=C-C);!$(C=C-C-[N,O,S]);!$(C=	Number of allylic oxidation sites excluding steroid dienone
[$(a-[CH3]),$(a-[CH2]-[CH3]),$(a-	Number of aryl methyl sites for hydroxylation
[$(N(-[CH3])-C-[$(C~O),$(C-a),$(	Number of XCCNR groups
[$([N&R1]1(-C)CCC1),$([N&R1]1(	Number of tert-alicyclic amines (no heteroatoms, not quinine-like bridged N)
C[NH1]C(=O)OC	Number of alkyl carbamates (subject to hydrolysis)
[$([CX3](=[OX1])(C)([c,C]));!$([CX	Number of ketones excluding diaryl, a,b-unsat. dienones, heteroatom on Calpha

IRFMN MGs
steroid scaffold
five_ring_hetero
six_ring_hetero
benzene-CH2
heteroatoms (O or S) bonded to a 6-atoms heterocyclic compound
heteroatoms (O or S) bonded to a 5-atoms heterocyclic compound
catechol, resorcinol, and hydroquinone
benzaldehyde
aromatic ring with OR sostituent
aromatic ring with R sostituent
ortho and para substitution with OH or OR
Ketone as ring substituent
benzoic acid or ester on aromatic ring
C bounded to three aromatic rings
Cl, Br bounded to aromatic ring
At least 3 OH or OR in the same ring
sulfoxide
terminal CH2
primary OH
triple bond
ethere
Ar-OS(=O)2
Quaternary C
Imidothioesters
anhydrides
Number of alkyl carbamates (subject to hydrolysis)
Number of aniline
Number of aniline
charge +
charge -
nitro aliphatic
ketone aliphatic
ketone aromatic

[$(a-[NX3H2]),$(a-[NH1][NH2]),$	Number of N functional groups attached to aromatics
[$([OX2H1][CX4][CX4H2][NX3&R	Number of C(OH)CCN-Ctert-alkyl or C(OH)CCNcyclic
