# Supplementary material for: Virtual Extensive Read-Across: A New Open-Access Software for Chemical Read-Across and Its Application to the Carcinogenicity Assessment of Botanicals
Source: Molecules. 2022 Oct 5;27(19):6605. doi: 10.3390/molecules27196605 (PMC9570968; doi:10.3390/molecules27196605)
Supplement: Supplementary file 1 [file molecules-27-06605-s001.zip › supplementary/molecules-1903480-supplementary2.pdf]

Structural Alerts (SAs) for carcinogeniciy

SMARTS of SAs	Description
[!$([OH1,SH1])]C(=O)[Br,Cl,F,I]	SA1_gen. Acyl halide RC(=O)[Br,Cl,F,I], where R is not OH or SH
S([!$([OH1,SH1])])(=O)(=O)O([$(C([#1,Cl,Br,I,F])([#1,Cl,Br,I,F])([#1,Cl,Br,I,F])),$(C([#1,Cl,Br,I,F])([#1,Cl,Br,I,F])C([#1,Cl,Br,I,F])([#1,Cl,Br,I,F])([#1,Cl,Br,I,F])),$(C([#1,Cl,Br,I,F])([#1,Cl,Br,I,F])C([#1,Cl,Br,I,F])([#1,Cl,Br,I,F])C([#1,Cl,Br,I,F])([#1,Cl,Br,I,F])([#1,Cl,Br,I,F])),$(C([#1,Cl,Br,I,F])(C([#1,Cl,Br,I,F])([#1,Cl,Br,I,F])([#1,Cl,Br,I,F]))C([#1,Cl,Br,I,F])([#1,Cl,Br,I,F])([#1,Cl,Br,I,F])),$(C([#1,Cl,Br,I,F])([#1,Cl,Br,I,F])C([#1,Cl,Br,I,F])([#1,Cl,Br,I,F])C([#1,Cl,Br,I,F])([#1,Cl,Br,I,F])C([#1,Cl,Br,I,F])([#1,Cl,Br,I,F])([#1,Cl,Br,I,F])),$(C([#1,Cl,Br,I,F])([#1,Cl,Br,I,F])C([#1,Cl,Br,I,F])(C([#1,Cl,Br,I,F])([#1,Cl,Br,I,F])([#1,Cl,Br,I,F]))C([#1,Cl,Br,I,F])([#1,Cl,Br,I,F])([#1,Cl,Br,I,F])),$(C(C([#1,Cl,Br,I,F])([#1,Cl,Br,I,F])([#1,Cl,Br,I,F]))(C([#1,Cl,Br,I,F])([#1,Cl,Br,I,F])([#1,Cl,Br,I,F]))C([#1,Cl,Br,I,F])([#1,Cl,Br,I,F])([#1,Cl,Br,I,F])),$(C([#1,Cl,Br,I,F])(C([#1,Cl,Br,I,F])([#1,Cl,Br,I,F])([#1,Cl,Br,I,F]))C([#1,Cl,Br,I,F])([#1,Cl,Br,I,F])C([#1,Cl,Br,I,F])([#1,Cl,Br,I,F])([#1,Cl,Br,I,F])),$([CH2]c1ccccc1)])<br><br>P(=O)([!$([OH1,SH1])])(O([$(C([#1,Cl,Br,I,F])([#1,Cl,Br,I,F])([#1,Cl,Br,I,F])),$(C([#1,Cl,Br,I,F])([#1,Cl,Br,I,F])C([#1,Cl,Br,I,F])([#1,Cl,Br,I,F])([#1,Cl,Br,I,F])),$(C([#1,Cl,Br,I,F])([#1,Cl,Br,I,F])C([#1,Cl,Br,I,F])([#1,Cl,Br,I,F])C([#1,Cl,Br,I,F])([#1,Cl,Br,I,F])([#1,Cl,Br,I,F])),$(C([#1,Cl,Br,I,F])(C([#1,Cl,Br,I,F])([#1,Cl,Br,I,F])([#1,Cl,Br,I,F]))C([#1,Cl,Br,I,F])([#1,Cl,Br,I,F])([#1,Cl,Br,I,F])),$(C([#1,Cl,Br,I,F])([#1,Cl,Br,I,F])C([#1,Cl,Br,I,F])([#1,Cl,Br,I,F])C([#1,Cl,Br,I,F])([#1,Cl,Br,I,F])C([#1,Cl,Br,I,F])([#1,Cl,Br,I,F])([#1,Cl,Br,I,F])),$(C([#1,Cl,Br,I,F])([#1,Cl,Br,I,F])C([#1,Cl,Br,I,F])(C([#1,Cl,Br,I,F])([#1,Cl,Br,I,F])([#1,Cl,Br,I,F]))C([#1,Cl,Br,I,F])([#1,Cl,Br,I,F])([#1,Cl,Br,I,F])),$(C(C([#1,Cl,Br,I,F])([#1,Cl,Br,I,F])([#1,Cl,Br,I,F]))(C([#1,Cl,Br,I,F])([#1,Cl,Br,I,F])([#1,Cl,Br,I,F]))C([#1,Cl,Br,I,F])([#1,Cl,Br,I,F])([#1,Cl,Br,I,F])),$(C([#1,Cl,Br,I,F])(C([#1,Cl,Br,I,F])([#1,Cl,Br,I,F])([#1,Cl,Br,I,F]))C([#1,Cl,Br,I,F])([#1,Cl,Br,I,F])C([#1,Cl,Br,I,F])([#1,Cl,Br,I,F])([#1,Cl,Br,I,F])),$([CH2]c1ccccc1)]))O([$(C([#1,Cl,Br,I,F])([#1,Cl,Br,I,F])([#1,Cl,Br,I,F])),$(C([#1,Cl,Br,I,F])([#1,Cl,Br,I,F])C([#1,Cl,Br,I,F])([#1,Cl,Br,I,F])([#1,Cl,Br,I,F])),$(C([#1,Cl,Br,I,F])([#1,Cl,Br,I,F])C([#1,Cl,Br,I,F])([#1,Cl,Br,I,F])C([#1,Cl,Br,I,F])([#1,Cl,Br,I,F])([#1,Cl,Br,I,F])),$(C([#1,Cl,Br,I,F])(C([#1,Cl,Br,I,F])([#1,Cl,Br,I,F])([#1,Cl,Br,I,F]))C([#1,Cl,Br,I,F])([#1,Cl,Br,I,F])([#1,Cl,Br,I,F])),$(C([#1,Cl,Br,I,F])([#1,Cl,Br,I,F])C([#1,Cl,Br,I,F])([#1,Cl,Br,I,F])C([#1,Cl,Br,I,F])([#1,Cl,Br,I,F])C([#1,Cl,Br,I,F])([#1,Cl,Br,I,F])([#1,Cl,Br,I,F])),$(C([#1,Cl,Br,I,F])([#1,Cl,Br,I,F])C([#1,Cl,Br,I,F])(C([#1,Cl,Br,I,F])([#1,Cl,Br,I,F])([#1,Cl,Br,I,F]))C([#1,Cl,Br,I,F])([#1,Cl,Br,I,F])([#1,Cl,Br,I,F])),$(C(C([#1,Cl,Br,I,F])([#1,Cl,Br,I,F])([#1,Cl,Br,I,F]))(C([#1,Cl,Br,I,F])([#1,Cl,Br,I,F])([#1,Cl,Br,I,F]))C([#1,Cl,Br,I,F])([#1,Cl,Br,I,F])([#1,Cl,Br,I,F])),$(C([#1,Cl,Br,I,F])(C([#1,Cl,Br,I,F])([#1,Cl,Br,I,F])([#1,Cl,Br,I,F]))C([#1,Cl,Br,I,F])([#1,Cl,Br,I,F])C([#1,Cl,Br,I [illegible]	SA2_gen. Methyl, ethyl, propyl, butyl or benzyl esters of sulphonic or phosphonic acid. P(=O)(O)(O)R or S(=O)(O)(O)R where R is not S or O The alkyl chains can have halogen substituents
[CX4H2](N)([OX2H1])	SA3_gen. N-methylol derivativesH
[C;D1]=[C;D2]([Cl,F,Br,I])<br><br>[C;D1]=[C;D3]([Cl,F,Br,I])([C;!$(C=*);!$(C#*)])<br><br>[C;D2]([!Cl;!Br;!F;!I;!$(C=O)])=[C;D2]([Cl,F,Br,I])<br><br>[C;D2]([!Cl;!Br;!F;!I;!$(C=O)])=[C;D3]([Cl,F,Br,I])([C;!$(C=*);!$(C#*)])<br>[C;D3]([!Cl;!Br;!F;!I;!$(C=O)])([C;!$(C=*);!$(C#*)])=[C;D2]([Cl,F,Br,I])<br>[C;D3]([!Cl;!Br;!F;!I;!$(C=O)])([C;!$(C=*);!$(C#*)])=[C;D3]([Cl,F,Br,I])([C;!$(C=*);!$(C#*)])	SA4_gen. This alert contains halogenated olefins where at least one hydrogen or alkyl group is attached to each carbon atom
[F,Cl,Br,I][CX4H2][CX4H2][N,S][CX4H2][CX4H2][F,Cl,Br,I]	SA5_gen. S or N mustard
[O,S]=C1[O,S]CC1<br>O=S1(=O)(CCCO1)	SA6_gen. propiolactones and propiosultones
C1[O,N]C1	SA7_gen. epoxides and aziridines
[$([CX4!H0;R0]);!$(C([#1,C])=[O,C]);!$([CX4H2]([F,Cl,Br,I])[CX4H2][N,S][CX4H2][CX4H2][F,Cl,Br,I]);!$(COP(O)(=O));!$(COS(=O)(=O));!$(CCOP(O)(=O));!$(CCOS(=O)(=O));!$(CCCOP(O)(=O));!$(CCCOS(=O)(=O));!$(CCCCOP(O)(=O));!$(CCCCOS(=O)(=O));!$(CCCCCOP(O)(=O));!$(CCCCCOS(=O)(=O));!$(CCCCCCOP(O)(=O));!$(CCCCCCOS(=O)(=O))][Cl,Br,I]	SA8_gen. This alert contains non tertiary aliphatic halogens
O=[NX2]OC	SA9_gen
[#6][$([CX3H1]);!$(CC=C)](=O)	SA11_gen. Aliphatic and aromatic aldehydes
O=[#6]1[#6]=,:[#6][#6](=O)[#6]=,:[#6]1<br>O=[#6]1[#6]=,:[#6][#6]=,:[#6][#6]1(=O)	SA12_gen. Quinones
[N+0]!@;-[N+0](=[!O;!N])<br><br>[N+0]([#1,*])!@;-[N+0]([#1,*])<br><br>[NX3H]-[NX3H2]	SA13_gen. This applies to molecules that contain a NN group not in a ring, and not NN=O
[C,#1]N=[NX2][C,#1]<br>[$(C=[N+]=[N-]);!$(C=[N+]=[N-]=N);!$(C=[N+]=[N-]N)]<br>C=[$(N=N);!$(N=N=N);!$(N=NN)]<br>CN=NO	SA14_gen. Aliphatic azo and azoxy
[NX2]=C=[O,S]	SA15_gen. Isocyanate and isothiocyanate groups
[NH2;D1]C(=[O,S])[O,S][CX4]<br>[NH;D2]([C;!$(C=*);!$(C#*)])C(=[O,S])[O,S][CX4]<br>[N;D3]([C;!$(C=*);!$(C#*)])C(=[O,S])[O,S][CX4]	SA16_gen. Alkyl carbamate and thiocarbamate
counter	SA18_gen. Polycyclic Aromatic Hydrocarbons, with three or more fused rings
counter	SA19_gen. Heterocyclic Polycyclic Aromatic Hydrocarbons (3 or more fused rings)
[C,c]N[NX2;v3]=O	SA21_gen. Alkyl and aryl N-nitroso groups
[N]=[N]-[N]<br>[N]=[N]=[N]	SA22_gen. Azide and triazene groups
[C!r][NH1]N(=O)O<br>[C!r]N(A)N(=O)O	SA23_gen. Aliphatic N-nitro
[!$([#6](=O)[!O]),#1][C!H0;!R]([!$([#6](=O)[!O]),#1])!@;=[C!H0;!R]O[#6]	SA24_gen. unsaturated alkoxy
[a;!$(a(a[A;!#1])(a[A;!#1]));!$(aa[CX3](=O)[OX2H1]);!$(aa[SX4](=[OX1])(=[OX1])([O]));!$(aaa[SX4](=[OX1])(=[OX1])([O]));!$(aaaa[SX4](=[OX1])(=[OX1])([O]));!$(aaaaa[SX4](=[OX1])(=[OX1])([O]));!$(aaaaaa[SX4](=[OX1])(=[OX1])([O]))]!@[$([NX2]=O)]	SA25_gen. Aromatic nitroso group
[n+]!@[O-]	SA26_gen. Aromatic ring N-oxide

[a;!$(a(a[A;!#1;!H])(a[A;!#1;!H]));!$(aa[CX3](=O)[OX2H1]);!$(aa[SX4](=[OX1])(=[OX1])([OX2H1]));!$(aaa[SX4](=[OX1])(=[OX1])([OX2H1]));!$(aaaa[SX4](=[OX1])(=[OX1])([OX2H1]));!$(aaaaa[SX4](=[OX1])(=[OX1])([OX2H1]));!$(aaaaaa[SX4](=[OX1])(=[OX1])([OX2H1]))]([$([N+]([O-])=O)])	SA27_gen. Nitro aromatic,SA27 Nitro aromatic: Nitro aromatic. However: Aromatic nitro groups with ortho-disubstitution or with a carboxylic acid substituent in ortho position should be excluded. Please note that a molecule like this **CC1=CC=CC(=C1[N+](=O)[O-])[N+](=O)[O-]** should be included in the alert: one of the two nitro groups is ortho disubstituted, but the other one is ortho-monosubstituted. Also the following molecule **CC2=CC=CC(CCC1=CC=CC(=C1)[N+](=O)[O-])=C2[N+](=O)[O-]** Should fire the alert (one nitro group is ortho disubstituted, but the other is not). If a sulfonic acid group (-SO3H) is present on the ring that contains also the nitro group, the substance should be excluded
[a;!$(a(a[A;!#1])(a[A;!#1]));!$(aa[CX3](=O)[OX2H1]);!$(aa[SX4](=[OX1])(=[OX1])([O]));!$(aaa[SX4](=[OX1])(=[OX1])([O]));!$(aaaa[SX4](=[OX1])(=[OX1])([O]));!$(aaaaa[SX4](=[OX1])(=[OX1])([O]));!$(aaaaaa[SX4](=[OX1])(=[OX1])([O]))]!@[$([N+0;H2;D1]),$([N+0;H1;D2][OH;D1]),$([N+0;H0;D3]([OH;D1])C),$([N+0;H1;D2]OC=O),$([N+0;H0;D3](C)OC=O)]	SA28_gen. Primary aromatic amine, hydroxyl amine and its derived esters (with restrictions). SA28 Primary aromatic amine, hydroxyl amine and its derived esters (with restrictions): Primary aromatic amine, hydroxyl amine and its derived esters (with restrictions). However: Aromatic amino groups with ortho disubstitutions or with a carboxylic acid substituent in ortho position are excluded. If a sulfonic acid group (-SO3H) is present on the ring that contains also the amino group, the substance should be excluded from the alert. The following structures should also be included: O=C=NC1=CC=CC=C1 and C([H])([H])=NC1=CC=CC=C1. The possibility that the Nitrogen atom of hydroxyl amine is part of a cycle, should be excluded
aN=C=O<br>aN=[CH2]<br>aN=[CH2]	SA28_gen. Primary aromatic amine, hydroxyl amine and its derived esters (with restrictions). SA28 Primary aromatic amine, hydroxyl amine and its derived esters (with restrictions): Primary aromatic amine, hydroxyl amine and its derived esters (with restrictions). However: Aromatic amino groups with ortho disubstitutions or with a carboxylic acid substituent in ortho position are excluded. If a sulfonic acid group (-SO3H) is present on the ring that contains also the amino group, the substance should be excluded from the alert. The following structures should also be included: O=C=NC1=CC=CC=C1 and C([H])([H])=NC1=CC=CC=C1. The possibility that the Nitrogen atom of hydroxyl amine is part of a cycle, should be excluded
[a;!$(a(a[A;!#1])(a[A;!#1]));!$(aa[CX3](=O)[OX2H1]);!$(aa[SX4](=[OX1])(=[OX1])([O]));!$(aaa[SX4](=[OX1])(=[OX1])([O]));!$(aaaa[SX4](=[OX1])(=[OX1])([O]));!$(aaaaa[SX4](=[OX1])(=[OX1])([O]));!$(aaaaaa[SX4](=[OX1])(=[OX1])([O]))]!@[$([NX3;v3]([#1,CH3])([CH3])),$([NX3;v3]([#1,CH3])([CH2][CH3])),$([NX3;v3]([CH2][CH3])([CH2][CH3]))]	SA28bis_gen. Mono- or di- methyl or ethyl aromatic amines, are included. SA28bis Aromatic mono- and dialkylamine: Mono- or di- methyl or ethyl aromatic amines, are included. However:Aromatic amino groups with ortho-disubstitution or with a carboxylic acid substituent in ortho position should be excluded. If a sulfonic acid group (-SO3H) is present on the ring that contains also the amino group, the substance should be excluded from the alert
[a;!$(a(a[A;!#1])(a[A;!#1]));!$(aa[CX3](=O)[OX2H1]);!$(aa[SX4](=[OX1])(=[OX1])([O]));!$(aaa[SX4](=[OX1])(=[OX1])([O]));!$(aaaa[SX4](=[OX1])(=[OX1])([O]));!$(aaaaa[SX4](=[OX1])(=[OX1])([O]));!$(aaaaaa[SX4](=[OX1])(=[OX1])([O]))]!@[$([NX3;v3]([#1,CH3])C(=O)([#1,CH3]))]	'SA28ter_gen. Aromatic N-acyl amine
a[N]=[N]a<br>[$([N](a)=[N]a);!$([N](a:aS(=[OD1])(=[OD1])[OD1])=[N]a:aS(=[OD1])(=[OD1])[OD1]);!$([N](a:aS(=[OD1])(=[OD1])[OD1])=[N]a:a:aS(=[OD1])(=[OD1])[OD1]);!$([N](a:aS(=[OD1])(=[OD1])[OD1])=[N]a:a:a:aS(=[OD1])(=[OD1])[OD1]);!$([N](a:aS(=[OD1])(=[OD1])[OD1])=[N]a:a:a:a:aS(=[OD1])(=[OD1])[OD1]);!$([N](a:aS(=[OD1])(=[OD1])[OD1])=[N]a:a:a:a:a:aS(=[OD1])(=[OD1])[OD1]);!$([N](a:aS(=[OD1])(=[OD1])[OD1])=[N]a:a:a:a:a:a:aS(=[OD1])(=[OD1])[OD1]);!$([N](a:a:aS(=[OD1])(=[OD1])[OD1])=[N]a:aS(=[OD1])(=[OD1])[OD1]);!$([N](a:a:aS(=[OD1])(=[OD1])[OD1])=[N]a:a:aS(=[OD1])(=[OD1])[OD1]);!$([N](a:a:aS(=[OD1])(=[OD1])[OD1])=[N]a:a:a:aS(=[OD1])(=[OD1])[OD1]);!$([N](a:a:aS(=[OD1])(=[OD1])[OD1])=[N]a:a:a:a:aS(=[OD1])(=[OD1])[OD1]);!$([N](a:a:aS(=[OD1])(=[OD1])[OD1])=[N]a:a:a:a:a:aS(=[OD1])(=[OD1])[OD1]);!$([N](a:a:aS(=[OD1])(=[OD1])[OD1])=[N]a:a:a:a:a:a:aS(=[OD1])(=[OD1])[OD1]);!$([N](a:a:a:aS(=[OD1])(=[OD1])[OD1])=[N]a:aS(=[OD1])(=[OD1])[OD1]);!$([N](a:a:a:aS(=[OD1])(=[OD1])[OD1])=[N]a:a:aS(=[OD1])(=[OD1])[OD1]);!$([N](a:a:a:aS(=[OD1])(=[OD1])[OD1])=[N]a:a:a:aS(=[OD1])(=[OD1])[OD1]);!$([N](a:a:a:aS(=[OD1])(=[OD1])[OD1])=[N]a:a:a:a:aS(=[OD1])(=[OD1])[OD1]);!$([N](a:a:a:aS(=[OD1])(=[OD1])[OD1])=[N]a:a:a:a:a:aS(=[OD1])(=[OD1])[OD1]);!$([N](a:a:a:aS(=[OD1])(=[OD1])[OD1])=[N]a:a:a:a:a:a:aS(=[OD1])(=[OD1])[OD1]);!$([N](a:a:a:a:aS(=[OD1])(=[OD1])[OD1])=[N]a:aS(=[OD1])(=[OD1])[OD1]);!$([N](a:a:a:a:aS(=[OD1])(=[OD1])[OD1])=[N]a:a:aS(=[OD1])(=[OD1])[OD1]);!$([N](a:a:a:a:aS(=[OD1])(=[OD1])[OD1])=[N]a:a:a:aS(=[OD1])(=[OD1])[OD1]);!$([N](a:a:a:a:aS(=[OD1])(=[OD1])[OD1])=[N]a:a:a:a:aS(=[OD1])(=[OD1])[OD1]);!$([N](a:a:a:a:a[illegible]	SA29_gen. Aromatic diazo
O=c1ccc2ccccc2(o1)<br>O=C1C=Cc2ccccc2O1	SA30_gen. Coumarins and Furocoumarins
C12CCCN1CC=C2	SA37_gen. Genotoxic mechanism. Pyrrolizidine Alkaloids
c1ccccc1C[C;!R]=C	SA38_gen. Genotoxic mechanism. Alkenylbenzenes: Alkenylbenzenes
C[C@@]12CCC3c4c(CCC3C1CC[C@H]2O)cc(O)cc4	SA39_gen_and_nogen. Mixed genotoxic and non genotoxic mechanism. Steroidal estrogens: Steroidal estrogens
[!a,#1;!$(C1(=O)C=CC(=O)C=C1)][#6]([!a,#1;!$(C1(=O)C=CC(=O)C=C1)])!:;=[#6][#6](=O)[!O;!$([#6]1:,=[#6][#6](=O)[#6]:,=[#6][#6](=O)1)]<br>[$([#6]);!$([#6]1C(=O)[#6]:,=[#6]C(=O)[#6]:,=1);!$([#6]C!=;-[C;R]!=;-[C;R]!=;-[C;R]!=;-[C;R]!=;-[C;R])]!:;=[$([#6]);!$(C=C[a])][$([#6]);!$([#6]-O);!$(C1(=O)[#6]:,=[#6]C(=O)[#6]:,=[#6]1)](=O)	SA10_gen. ?,? unsaturated carbonyls
[#7X3][#6](=[SX1])[!$([O,S][CX4])!$([OH,SH])!$([O-,S-])]	SA17_nogen. Thiocarbonyl (Nongenotoxic carcinogens)

[c;!$(c[N+](=O)[O]);!$(c[N]([#1,C])([#1,C]));!$(cN([OX2H])([#1,C]));!$(cN([#1,C])OC=O);!$(c[NX3v3]([#1,CH3])([#1,CH3]));!$(c[NX3v3]([#1,CH3])([CH2][CH3]));!$(c[NX3v3]([CH2][CH3])([CH2][CH3]));!$(cNC(=O)[#1,CH3]);!$(cN=[N]a);!$(c!@[cR1r6]1ccccc1);!$(c!@*!@c1ccccc1);!$([R2])]1[c;!$(c[N+](=O)[O]);!$(c[N]([#1,C])([#1,C]));!$(cN([OX2H])([#1,C]));!$(cN([#1,C])OC=O);!$(c[NX3v3]([#1,CH3])([#1,CH3]));!$(c[NX3v3]([#1,CH3])([CH2][CH3]));!$(c[NX3v3]([CH2][CH3])([CH2][CH3]));!$(cNC(=O)[#1,CH3]);!$(cN=[N]a);!$(c!@[cR1r6]1ccccc1);!$(c!@*!@c1ccccc1);!$([R2])][c;!$(c[N+](=O)[O]);!$(c[N]([#1,C])([#1,C]));!$(cN([OX2H])([#1,C]));!$(cN([#1,C])OC=O);!$(c[NX3v3]([#1,CH3])([#1,CH3]));!$(c[NX3v3]([#1,CH3])([CH2][CH3]));!$(c[NX3v3]([CH2][CH3])([CH2][CH3]));!$(cNC(=O)[#1,CH3]);!$(cN=[N]a);!$(c!@[cR1r6]1ccccc1);!$(c!@*!@c1ccccc1);!$([R2])][c;!$(c[N+](=O)[O]);!$(c[N]([#1,C])([#1,C]));!$(cN([OX2H])([#1,C]));!$(cN([#1,C])OC=O);!$(c[NX3v3]([#1,CH3])([#1,CH3]));!$(c[NX3v3]([#1,CH3])([CH2][CH3]));!$(c[NX3v3]([CH2][CH3])([CH2][CH3]));!$(cNC(=O)[#1,CH3]);!$(cN=[N]a);!$(c!@[cR1r6]1ccccc1);!$(c!@*!@c1ccccc1);!$([R2])][c;!$(c[N+](=O)[O]);!$(c[N]([#1,C])([#1,C]));!$(cN([OX2H])([#1,C]));!$(cN([#1,C])OC=O);!$(c[NX3v3]([#1,CH3])([#1,CH3]));!$(c[NX3v3]([#1,CH3])([CH2][CH3]));!$(c[NX3v3]([CH2][CH3])([CH2][CH3]));!$(cNC(=O)[#1,CH3]);!$(cN=[N]a);!$(c!@[cR1r6]1ccccc1);!$(c!@*!@c1ccccc1);!$([R2])]c1([Cl,Br,F,I;!$([Cl,Br,I,F]cc[Cl,Br,I,F]);!$([Cl,Br,I,F]ccc[Cl,Br,I,F]);!$([Cl,Br,I,F]c1c([OX2H])c([OX2H])c([OX2H])cc1);!$([Cl,Br,I,F]c1c([OX2H])c([OX2H])cc([OX2H])c1);!$([Cl,Br,I,F]c1c([OX2H])c([OX2H])ccc1([OX2H]));!$([Cl,Br,I,F]c1c([OX2H])cc([OX2H])c([OX2H])c1);!$([Cl,Br,I,F]c1c([OX2H])cc([OX2H])cc1([OX2H]));!$([Cl,Br,I,F]c1cc([OX2H])c([OX2H])c([OX2H][illegible]	SA31a_nogen. Halogenated benzene (Nongenotoxic carcinogens)
[Cl,Br,F,I]c1ccc2ccccc2(c1)<br><br>[Cl,Br,F,I]c1ccc(cc1)!@c2ccc(cc2)[Cl,Br,F,I]<br><br>c1cc(ccc1[!R]c2ccc(cc2)[Cl,Br,F,I])[Cl,Br,F,I]	SA31b_nogen. Halogenated PAH (naphthalenes, biphenyls, diphenyls) (Nongenotoxic carcinogens)
c1ccc2Oc3cc(ccc3(Oc2(c1)))[Cl,Br,F,I]	SA31c_nogen. Halogenated dibenzodioxins (Nongenotoxic carcinogens)
c1(OC(C)(C)C(=O)O)ccc([#6,#17])cc1<br><br>c1(OCC(=O)[O;H0])cc(Cl)c(Cl)cc1<br><br>c1(OCC(=O)[O;H0])c(Cl)cc(Cl)cc1	SA40_nogen. Nongenotoxic mechanism. Substituted phenoxyacid: Substituted phenoxyacid
[C;!R&$(C([C;!R])([C;!R])[C;!R][C;!R])&!$(CCCCCCCCCCCC)][C;!R&$(C[OX2;!R]),$(C(=O)[OX2;!R])]	SA41_nogen. Nongenotoxic mechanism. Substituted n-alkylcarboxylic acids: Substituted n-alkylcarboxylic acids
O=C(O)c1ccccc1C(=O)O<br><br>O=C(O)[CX4;!R][CX4;!R][CX4;!R][CX4;!R]C(=O)O	SA42_nogen. Nongenotoxic mechanism. Phthalate diesters and monoesters: Phthalate diesters and monoesters
CC(F)(F)C(F)(F)C(F)(F)C(F)(F)C(F)(F)F	SA43_nogen. Nongenotoxic mechanism. SA43 Perfluorooctanoic acid (PFOA): Perfluorooctanoic acid (PFOA)
[Cl,F][C;!$(Cc)]=C([Cl,F])[Cl,F]<br><br><br>Cl[C;!$(Cc)]=C(Cl)Cl<br><br><br>[Cl,F]C#C[Cl,F]	SA44_nogen. Nongenotoxic mechanism. SA44 Trichloro (or fluoro) ethylene and Tetrachloro (or fluoro) ethylene: Trichloro (or fluoro) ethylene and Tetrachloro (or fluoro) ethylene
OCc1c[nH]c2ccccc12	SA45_nogen. Ngenotoxic mechanisms. Indole-3-carbinol: Indole-3-carbinol
Clc1c(Cl)c(Cl)c(Cl)c(Cl)c1O	SA46_nogen. Nongenotoxic mechanism. Pentachlorophenol: Pentachlorophenol
Oc2ccccc2c1ccccc1<br><br>Oc1c(c2ccccc2)cccc1	SA47_nogen. Nongenotoxic mechanisms. O-phenylphenol: O-phenylphenol
Oc1cc(O)c2C(=O)C(O)=C(Oc2c1)c3ccc(O)c(O)c3	SA48_nogen. Nongenotoxic mechanisms. SA48 Quercetin-type flavonoids: Quercetin-type flavonoids
n1c[nH]cc1<br><br>n2c1ccccc1nc2	SA49_nogen. Nongenotoxic mechanism. Imidazole and benzimidazole: Imidazole and benzimidazole
[#6]1[#6](=O)[#7][#6](=O)[#6]1	SA50_nogen. Nongenotoxic mechanism. Dicarboximide: Dicarboximide
[CX4H3]c1cccc([CX4H3])n1	SA51_nogen. Nongenotoxic mechanism. Dimethylpyridine: Dimethylpyridine
[As,Cu,Cr,Hg,Co]	SA52_nogen. Nongenotoxic mechanism. Metals, oxidative stress: Metals, oxidative stress
c1cc(N)ccc1S(=O)(=O)N<br><br>c1cc(S)ccc1S(=O)(=O)N<br><br>c1ccccc1S(=O)(=O)[N;-1]<br><br>c1cc(C)ccc1S(=O)(=O)O	SA53_nogen. Nongenotoxic mechanism. SA53 Benzensulfonic ethers: Benzensulfonic ethers
[C;!$(C(C)(C))]1Oc2ccccc2O1	SA54_nogen. Nongenotoxic mechanism. 1,3-Benzodioxoles: 1,3-Benzodioxoles
c1cc(O)ccc1OC(C)C(=O)[O;!$(OCCCC)]	SA55_nogen. Nongenotoxic mechanisms. Phenoxy herbicides: Phenoxy herbicides
[C;H1,!$(CCOP)&!$(CCP)&!$(CF)&!$(CN)&!$(C[CH3])](Cl)(Cl)(Cl)	SA56_nogen. Nongenotoxic mechanism
[N+0;!R;!$(N=*);!$(N#*)][C+0;!R;!$(C=*);!$(C#*)][C+0;!R;!$(C=*);!$(C#*)][N+0;!R;!$(N=*);!$(N#*)]Cc1c2aaaac2aaa1<br><br><br>[N+0;!R;!$(N=*);!$(N#*)][C+0;!R;!$(C=*);!$(C#*)][C+0;!R;!$(C=*);[N+0;!R;!$(N=*);!$(N#*)][C+0;!R;!$(C=*);!$(C#*)][C+0;!R;!$(C=*);!$(C#*)][N+0;!R;!$(N=*);!$(N#*)]Cc1c2[A][A]=[A][A]c2aaa1<br><br>[N+0;!R;!$(N=*);!$(N#*)][C+0;!R;!$(C=*);!$(C#*)][C+0;!R;!$(C=*);!$(C#*)][N+0;!R;!$(N=*);!$(N#*)]Cc1c2[A][A]=[A]c2aaa1<br><br>[N+0;!R;!$(N=*);!$(N#*)][C+0;!R;!$(C=*);!$(C#*)][C+0;!R;!$(C=*);!$(C#*)][N+0;!R;!$(N=*);!$(N#*)]Cc1c2Cc3aaaac3c2aaa1<br><br>[N+0;!R;!$(N=*);!$(N#*)][C+0;!R;!$(C=*);!$(C#*)][C+0;!R;!$(C=*);!$(C#*)][N+0;!R;!$(N=*);!$(N#*)]Cc1c2c3aaaac3Cc2aaa1	SA57 DNA Intercalating Agents with a basic side chain: DNA intercalating agents are defined as those compounds that are able to insert partially or completely between adjacent DNA base pairs
