# Supplementary material for: Virtual Extensive Read-Across: A New Open-Access Software for Chemical Read-Across and Its Application to the Carcinogenicity Assessment of Botanicals
Source: Molecules. 2022 Oct 5;27(19):6605. doi: 10.3390/molecules27196605 (PMC9570968; doi:10.3390/molecules27196605)
Supplement: Supplementary file 1 [file molecules-27-06605-s001.zip › supplementary/molecules-1903480-supplementary3.pdf]

Molecular groups prevalence in carcinogenicity dataset

MGs	Active	NoActive	Prevalence%	Tox
Al_COO	59	109	35	Neutral
Al_OH	269	355	43	Neutral
ArN	150	62	71	Tox
Ar_OH	1	3	25	NoTox
C_S	10	27	27	NoTox
HOCCN	8	9	47	Neutral
Imine	20	28	42	Neutral
NH0	759	433	64	Neutral
NH1	133	176	43	Neutral
NH2	92	152	38	Neutral
N_O	29	8	78	Tox
Ndealkylat	37	60	38	Neutral
Ndealkylat	11	30	27	NoTox
Nhpyrrole	38	38	50	Neutral
SH	0	4	0	NoTox
aldehyde	17	8	68	Tox
alkyl_carba	1	1	50	Neutral
alkyl_halid	309	158	66	Tox
allylic_oxic	208	237	47	Neutral
amide	174	146	54	Neutral
amidine	6	8	43	Neutral
aniline	140	96	59	Neutral
aryl_methy	179	118	60	Neutral
azide	1	0	100	Tox
azo	47	20	70	Tox
barbitur	1	3	25	NoTox
benzene	317	242	57	Neutral
benzodiaze	0	6	0	NoTox
bicyclic	663	385	63	Neutral
diazo	2	0	100	Tox
dihydropyr	4	8	33	NoTox
epoxide	16	11	59	Neutral
ester	100	129	44	Neutral
ether	175	276	39	Neutral
furan	42	7	86	Tox
guanido	10	19	34	NoTox
hdrzine	49	13	79	Tox
hdrzone	31	13	70	Tox
imidazole	31	24	56	Neutral
imide	11	18	38	Neutral
isocyan	6	0	100	Tox
isothiocyar	1	2	33	NoTox
ketone	111	87	56	Neutral
ketone_To	65	65	50	Neutral

lactam	0	4	0	NoTox
lactone	24	21	53	Neutral
methoxy	91	88	51	Neutral
morpholin	10	5	67	Tox
nitrile	11	19	37	Neutral
nitro_arom	55	51	52	Neutral
nitroso	164	27	86	Tox
oxazole	0	2	0	NoTox
oxime	3	5	38	Neutral
para_hydr	102	90	53	Neutral
phenol	98	114	46	Neutral
phenol_no	93	109	46	Neutral
phos_acid	7	4	64	Neutral
phos_ester	7	3	70	Tox
piperzine	17	14	55	Neutral
priamide	0	0	NA	No Data
prisulfonar	0	0	NA	No Data
pyridine	58	54	52	Neutral
quatN	46	73	39	Neutral
sulfide	14	23	38	Neutral
sulfonamd	11	20	35	Neutral
sulfone	4	3	57	Neutral
term_acety	6	2	75	Tox
tetrazole	2	1	67	Tox
thiazole	19	9	68	Tox
thiocyan	0	1	0	NoTox
thiophene	12	6	67	Tox
unbrch_alk	152	205	43	Neutral
urea	52	31	63	Neutral
heteroator	37	46	45	Neutral
heteroator	58	141	29	NoTox
steroid	23	8	74	Tox
benzCH2	140	116	55	Neutral
biphenol	21	28	43	Neutral
Ar_OR	133	128	51	Neutral
Ar_R	253	273	48	Neutral
op_diphen	33	43	43	Neutral
Ar_COR	28	33	46	Neutral
Ar_COO_R	29	35	45	Neutral
C_3phenol	5	2	71	Tox
Ar_Cl_Br	163	176	48	Neutral
Ring_3OH_	8	12	40	Neutral
Sulfoxide	15	6	71	Tox
CH2_Term	63	38	62	Neutral
triple_bon	23	22	51	Neutral
Ar_COO	13	20	39	Neutral
Ar_OSO2	2	3	40	Neutral

CC4	208	205	50	Neutral
Imidothioe	0	0	NA	No Data
anhydrides	0	2	0	NoTox
carbamate	10	8	56	Neutral
aniline_ter	112	41	73	Tox
charge+	106	104	50	Neutral
charge-	107	104	51	Neutral
nitro_aliph	12	10	55	Neutral
ketone_ali	62	50	55	Neutral
Ar_ketone	22	29	43	Neutral
